# Maternal Weight Intervention in the Perinatal Period Improves Liver Health in the Offspring of Mothers with Obesity

**DOI:** 10.3390/nu16010109

**Published:** 2023-12-28

**Authors:** Amanda R. Purcell, Natassia Rodrigo, Qinghua Cao, Olivia Joseph, Anthony J. Gill, Sonia Saad, Carol A. Pollock, Sarah J. Glastras

**Affiliations:** 1 Kolling Institute of Medical Research, St Leonards, NSW 2065, Australia; 2 North Precinct, Sydney Medical School, University of Sydney, Sydney, NSW 2065, Australia; 3 Department of Diabetes, Endocrinology and Metabolism, Royal North Shore Hospital, St Leonards, NSW 2065, Australia; 4 Department of Diabetes and Endocrinology, Nepean Hospital, Sydney, NSW 2747, Australia; 5 NSW Health Pathology, Department of Anatomical Pathology, Royal North Shore Hospital, St Leonards, NSW 2065, Australia

**Keywords:** metabolic-associated fatty liver disease (MAFLD), liraglutide, inflammation, oxidative stress, fibrosis

## Abstract

Early-life exposure to maternal obesity predisposes offspring to metabolic-associated fatty liver disease (MAFLD). This study aimed to determine if peripartum weight loss, either through dietary intervention or pharmacological intervention, improved adverse liver health outcomes in the offspring of mothers with obesity. C57Bl/6 dams were fed a chow diet or a high-fat diet (HFD) for 8 weeks. HFD-fed mice either continued HFD, transitioned to a chow diet, or were administered liraglutide for 4 weeks. Pregnancy was induced following a one-week washout of liraglutide during which all animals remained on their respective diets. A proportion of HFD-fed mice transitioned to a chow diet during pregnancy. All offspring were weaned to the HFD. Offspring anthropometric, metabolic, and hepatic outcomes were assessed at postnatal week 12. The offspring of mothers with obesity had phenotypic changes consistent with MAFLD. The offspring of mothers that had weight loss with perinatal dietary intervention had reduced insulin resistance (*p* < 0.001) and hepatic expression of markers of inflammation (*p* < 0.001), oxidative stress (*p* < 0.05), and fibrosis (*p* < 0.05). A similar phenotype was observed in the offspring of mothers with pre-pregnancy weight loss via liraglutide despite ongoing consumption of the HFD during pregnancy. All methods and timing of maternal weight intervention were effective at ameliorating adverse liver effects in the offspring.

## 1. Introduction

Metabolic-associated fatty liver disease (MAFLD) refers to a range of conditions that are characterised by hepatic fat accumulation [1] and is the leading cause of chronic liver disease, affecting 25% of the global population [2]. MAFLD is strongly associated with obesity; it affects 75% of patients with a body mass index (BMI) > 30 kg/m^2^ compared to 16% of individuals with a normal BMI [3]. MAFLD ranges from simple steatosis, which is the intrahepatic accumulation of lipids, to its more serious stage, metabolic-associated steatohepatitis (MASH), which manifests as steatosis concurrent with inflammation, oxidative stress, and fibrosis in the liver [4].

The global incidence of obesity is climbing, and the proportion of women with obesity of childbearing age is increasing in parallel. Data from the US National Health and Nutrition Examination Survey showed more than 30% of American women aged 20–39 years have obesity [5]. Maternal obesity poses a health risk to both the mother and the child, increasing the chance of pregnancy-related complications, such as gestational diabetes and preeclampsia, and predisposing offspring to obesity and its comorbidities [6,7,8]. Children born to obese mothers have an increased risk of developing MAFLD in adulthood, a relationship that is directly related to maternal pre-pregnancy BMI and gestational weight gain (GWG) [9]. In preclinical rodent studies, an obesogenic maternal environment fostered the progression of MAFLD in the offspring, namely, steatosis, and the activation of pro-inflammatory and fibrotic pathways in the liver [10,11]. Efforts to address obesity in prospective mothers and promote healthy weight management in the perinatal period may reduce the risk of adverse liver health outcomes in the offspring.

Women with obesity are advised to implement lifestyle interventions to lose weight to improve fertility and pregnancy outcomes [12], yet it proves difficult to initiate and maintain weight loss [13]. Pharmacological weight loss agents may be a valuable aid for women with obesity. Glucagon-like peptide-1 receptor agonists (GLP-1RAs) promote satiety by acting on central hypothalamic neural pathways, resulting in meaningful weight loss [14]. Recently, it was shown that the pre-pregnancy administration of the GLP-1RA, liraglutide, induces weight loss, improves fertility, and improves maternal metabolic function in a rodent model of maternal obesity [15]. The effect on the offspring’s health has not yet been explored. This study aimed to ascertain the effectiveness of perinatal weight loss, either through diet modification or pharmacological intervention with GLP-1RA, liraglutide, in ameliorating the adverse outcomes for liver health in the offspring of mothers with obesity. We hypothesised that pre-pregnancy maternal weight loss, either with dietary intervention or pre-pregnancy liraglutide, would ameliorate the adverse liver health outcomes associated with MAFLD in the offspring of obese mothers.

## 2. Materials and Methods

### 2.1. Animal Experiments

**Figure 1:**
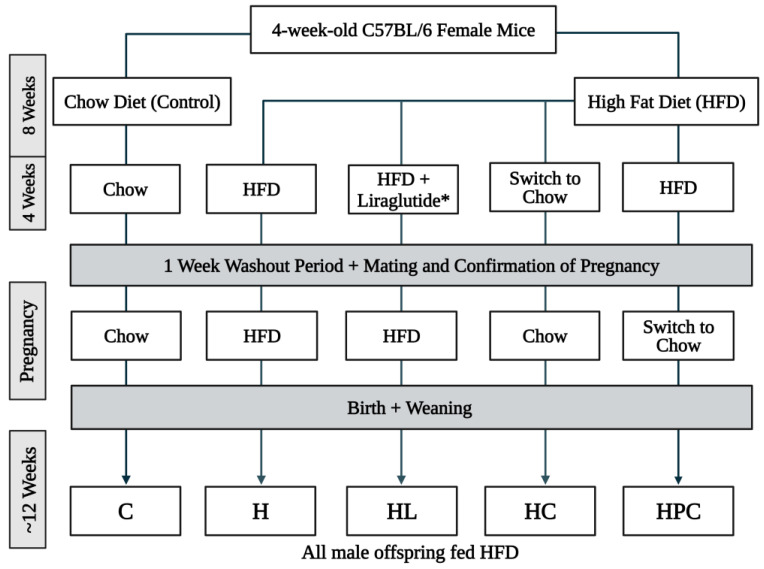
Study design schematic of mothers and their offspring in the mouse model. * The dose of liraglutide was given at incremental doses of 3 days of 0.1 mg/kg/day, 3 days of 0.2 mg/kg/day, and 0.3 mg/kg/day for the remainder of the four-week period.

An animal model of maternal obesity was conducted as previously described [15]. This project was approved by the Animal Care and Ethics Committee of the Northern Sydney Local Health District (Approval No. RESP/18/148). In brief, four-week-old female dams were fed either a standard chow diet (control) or a high-fat diet (HFD) (19 kJ/g, 43% fat, SF04-001; Specialty Feeds, Glen Forrest, Australia) for 8 weeks. After this time, HFD-fed dams either continued on the HFD, transitioned to chow diet, or were administered liraglutide via daily subcutaneous injection with incremental doses of 3 days of 0.1 mg/kg/day, 3 days of 0.2 mg/kg/day, and 0.3 mg/kg/day for the remainder of the 4 weeks. After a one-week washout of liraglutide, cohabitation with a male partner occurred, and pregnancy was then confirmed. The HFD group was then split during pregnancy; half of this group continued an HFD while the other half switched to a chow diet until delivery. All male offspring were weaned on an HFD until 12 weeks of age. The offspring groups consisted of eight males per group, and group names were given based on the mother’s diet, as summarised in Figure 1: HFD + liraglutide mother with HFD-fed offspring (HL), HFD-fed mother switched to chow in pre-pregnancy with HFD-fed offspring (HC), HFD-fed mother switched to chow during pregnancy with HFD-fed offspring (HPC), HFD-fed mother with HFD-fed offspring (H), and chow-fed mother with HFD-fed offspring (C). Fasting blood glucose measurements were taken after a 6 h fasting period using a glucometer (Accu-Chek^®^, Roche Diagnostics, Sydney, Australia) before the mice were sacrificed. The blood and liver were harvested and weighed. The liver samples were either snap frozen in liquid nitrogen or placed in 10% formalin solution for histological preparation.

### 2.2. Bioassays

The UltraSensitive Mouse Insulin ELISA Kit (Crystal Chem, Elk Grove Village, IL, USA) was used to quantify serum insulin, and the homeostatic model assessment of insulin resistance (HOMA-IR) score was calculated for each subject. Non-esterified fatty acids (NEFA) were measured using a NEFA kit (WAKO, Osaka, Japan). Serum monocyte chemoattractant protein-1 (MCP-1) was measured using an MCP-1 ELISA kit following the manufacturer’s instructions (Thermo Fisher Scientific, Scoresby, Australia). Serum quantification of aspartate transaminase (AST) and alanine transaminase (ALT) was obtained using the Architect C16000 Clinical Chemical Analyzer (Abbott Laboratories, Gurnee, IL, USA).

### 2.3. Quantitative Real-Time PCR

The RNeasy Plus Mini Kit (Qiagen, Redwood City, CA, USA) was used to extract total RNA from liver tissue. Then, 1000 ng of total RNA from each sample was reverse transcribed to cDNA using the iScript cDNA Synthesis Kit (Biorad, Hercules, CA, USA). Real time PCR (RT-PCR) was performed using the QuantStudio 12K Flex Real Time PCR System (Thermo Fisher Scientific, Scoresby, Australia) and the iTaq Universal SYBR Green Supermix (Bio-Rad, Hercules, CA, USA). The PCR primers used for 18S; collagen (COL)-I, -III, and -IV; MCP-1; superoxide dismutase 1 (SOD1); and sterol regulatory element binding protein-1c (SREBP-1c) are listed in Table 1. The results were normalised to 18S expression and expressed as the fold change relative to the control group.

### 2.4. Analysis of Structural Changes to the Liver

Liver sections fixed in formalin were embedded in paraffin and stained using picrosirius red to identify collagen networks. Frozen liver sections in OCT were stained with Oil Red O to assess hepatic lipid content. Six non-overlapping images were captured for each stain using a camera attached to the light microscope (Leica Application Suite, Leica, Germany). The staining area was quantified using Image J software (Java 1.8.0_345, National Institutes of Health, Bethesda, MD, USA). The data was averaged and expressed as the area percentage of staining.

### 2.5. Immunohistochemistry

Paraffin-embedded liver sections were de-paraffinized in xylene before rehydrating in graded concentrations of ethanol. Subsequent antigen retrieval was performed by incubating the sections in citrate buffer (99 °C, pH 6.0) for 20 min. The sections were then cooled at room temperature for 20 min, washed in phosphate-buffered saline (PBS, pH 7.4), and washed in running water. Endogenous peroxidase was quenched using 0.3% hydrogen peroxide (Sigma-Aldrich, Dublin, Ireland) before slides were blocked for 10 min with Protein Block Serum-Free (Dako, Glostrup, Denmark). The sections were incubated with primary antibodies against COL-IV (dilution 1:200, Abcam Ltd., Cambridge, MA, USA), 8-hydroxy-2’-deoxyguanosine (8-OHdg) (dilution 1:200, Cell Signalling Technology, Beverly, MA, USA), or MCP-1 (dilution 1:250, Abcam Ltd., Cambridge, MA, USA) at 4 °C overnight. They were then washed in PBS before undergoing incubation with the horseradish peroxidase anti-rabbit Envision system (Dako, Tokyo, Japan). Staining was developed with 3.3’-diaminobenzidine tetrahydrochloride (Dako, Tokyo, Japan) and then the slides were counterstained with Mayer’s haematoxylin followed by Scott’s Blue staining (Fronine, Riverstone, Australia). Six non-overlapping images were captured using a digital camera attached to the light microscope and quantified using Image J software (Java 1.8.0_345, National Institutes of Health, Bethesda, MD, USA).

### 2.6. Statistical Methods

All results are expressed as mean ± the standard error of the mean (SEM). One-way analysis of variance (ANOVA) was used, with post-hoc Fisher’s protected least-significant difference test (Prism 9.3.1 GraphPad Software, San Diego, CA, USA) with the following pre-specific group comparisons, to directly explore the hypotheses of this study: C vs. H, C vs. HL, C vs. HC, C vs. HPC, H vs. HL, H vs. HC, H vs. HPC, and HL vs. HC. Statistical significance was accepted at *p* < 0.05.

## 3. Results

### 3.1. Maternal HFD Causes Metabolic Disturbances in the Offspring at Postnatal Week 12

The offspring of HFD-fed mothers, regardless of maternal weight loss in the perinatal period, had increased body weights compared to the offspring of lean mothers (C vs. H, C vs. HL, C vs. HPC, *p* < 0.0001, and C vs. HC *p* < 0.01, Table 2). The offspring of mothers with pre-pregnancy weight loss via diet modification had significantly reduced body weight compared to the offspring of HFD-fed mothers and those administered liraglutide (H vs. HC *p* < 0.001, HL vs. HC *p* < 0.0001, Table 2). There was no significant difference in liver mass between the offspring groups (N.S, Table 2).

**Figure 2:**
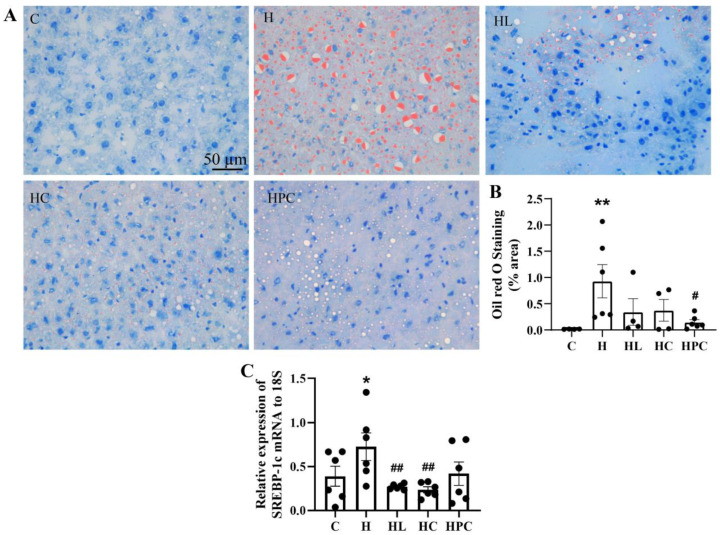
Lipid accumulation in twelve-week-old offspring. (**A**) Representative images of Oil Red O staining in the liver at 20× magnification, (**B**) area (%) of Oil Red O staining, and (**C**) mRNA expression of SREBP-1c in the liver (*n* = 6). Results are expressed as mean ± SEM. * *p* < 0.05, ** *p* < 0.01 when compared to C. # *p* < 0.05, ## *p* < 0.01 when compared to H.

Maternal obesity was associated with hyperinsulinemia and insulin resistance in the offspring, indicated by higher serum insulin levels and HOMA-IR scores, when compared to the offspring of lean mothers (serum insulin: C vs. H, *p* < 0.01, HOMA-IR: C vs. H, *p* < 0.001, Table 2). The offspring of mothers administered liraglutide or with dietary change in the perinatal period had significantly lowered serum insulin and HOMA-IR scores when compared to the offspring of mothers with obesity (serum insulin: H vs. HL, H vs. HPC, *p* < 0.01, H vs. HC, *p* < 0.001, HOMA-IR: H vs. HL, *p* < 0.01, H vs. HC, *p* < 0.0001, H vs. HPC, *p* < 0.001, Table 2). There were no differences in insulin levels or insulin resistance between the offspring of mothers with pre-pregnancy dietary intervention compared to those with liraglutide treatment (HC vs. HL, N.S, Figure 2B,C). There was no effect of maternal obesity and weight loss on blood glucose levels or NEFA levels between the offspring groups (N.S, Table 2). Maternal obesity and weight loss had no effect on AST or ALT levels between the offspring groups (N.S, Table 2).

### 3.2. Maternal Obesity Induces Steatosis in the Liver of Offspring

There was more lipid accumulation in the liver of the offspring born to obese mothers compared to the offspring of lean controls (C vs. H *p* < 0.01, Figure 2A,B). There was a trend towards smaller lipid droplet size and abundance in the offspring born to mothers with pre-pregnancy weight loss (either liraglutide or diet change), and significantly less lipid accumulation in the liver of offspring of mothers with weight loss during pregnancy (H vs. HPC *p* < 0.05, Figure 2A,B). There was no difference in lipid accumulation in the liver between the offspring of mothers with pre-pregnancy dietary intervention compared to those with liraglutide treatment (HC vs. HL, N.S, Figure 2A). 

There was higher SREBP-1c expression in the livers of offspring of mothers with obesity compared to the offspring of lean mothers (C vs. H, *p* < 0.05, Figure 2C). Expression of SREBP-1c mRNA in the liver was significantly reduced in the offspring of mothers with pre-pregnancy weight loss via liraglutide administration or diet modification compared to the offspring of mothers with obesity (H vs. HL, H vs. HC, *p* < 0.01, Figure 2C). 

There was no difference in the expression of SREBP-1c in the liver of the offspring born to HFD-fed mothers compared to the offspring of mothers with dietary intervention during pregnancy (H vs. HPC, N.S, Figure 2C). There was no difference in SREBP-1c expression between the offspring of pre-pregnancy liraglutide-treated versus pre-pregnancy diet switch groups (HC vs. HL, N.S, Figure 2C).

### 3.3. Maternal Obesity Increases Inflammation and Oxidative Stress in the Liver of Offspring Which Is Attenuated by Weight Loss in the Perinatal Period

**Figure 3:**
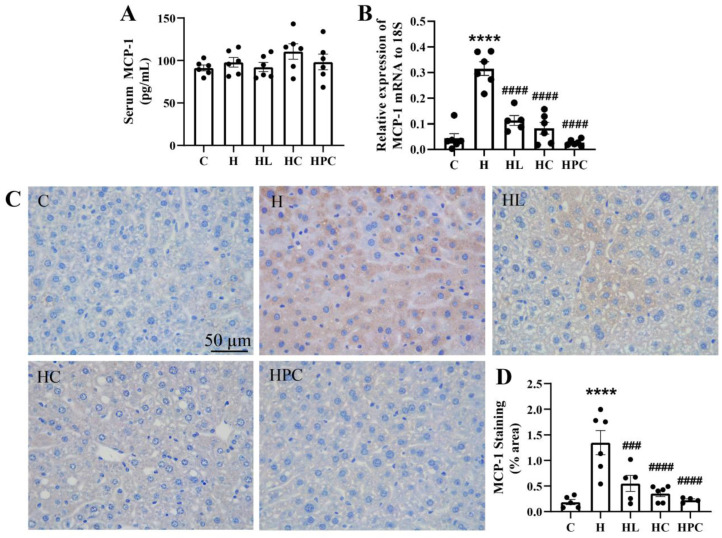
Inflammation in twelve-week-old offspring. (**A**) Serum MCP-1 levels (*n* = 6) and (**B**) mRNA expression in the liver of the inflammatory marker MCP-1 (*n* = 6). (**C**) Representative images of MCP-1 immunohistochemistry at 20× magnification and (**D**) area (%) of MCP-1 staining (*n* = 5–6). Results are expressed as mean ± SEM. **** *p* < 0.0001 when compared to C. ### *p* < 0.001, #### *p* < 0.0001 when compared to H.

Maternal obesity and perinatal weight intervention had no effect on the inflammatory serum cytokine, MCP-1, in the offspring (Figure 3A). However, there was a six-fold increase in the mRNA expression of MCP-1 in the liver of the offspring of mothers with obesity compared to the offspring of lean mothers (C vs. H *p* < 0.0001, Figure 3B). MCP-1 expression was significantly reduced in the liver of offspring of mothers with weight intervention in the perinatal period, through either liraglutide or diet change, compared to the offspring of HFD-fed mothers (H vs. HL, H vs. HC, H vs. HPC, *p* < 0.0001, Figure 3B).

The offspring of HFD-fed mothers also had elevated MCP-1 protein levels when compared to the offspring of lean mothers (C vs. H, *p* < 0.0001, Figure 3C,D). Analogous to MCP-1 mRNA expression, MCP-1 protein expression was significantly reduced in the offspring of mothers with weight intervention in the perinatal period compared to the offspring of mothers with obesity (H vs. HL, *p* < 0.001, H vs. HC, H vs. HPC, *p* < 0.0001, Figure 3C,D). There was no difference in hepatic MCP-1 gene or protein expression between the offspring of mothers with pre-pregnancy liraglutide, pre-pregnancy dietary intervention, or post-conception dietary switch.

**Figure 4:**
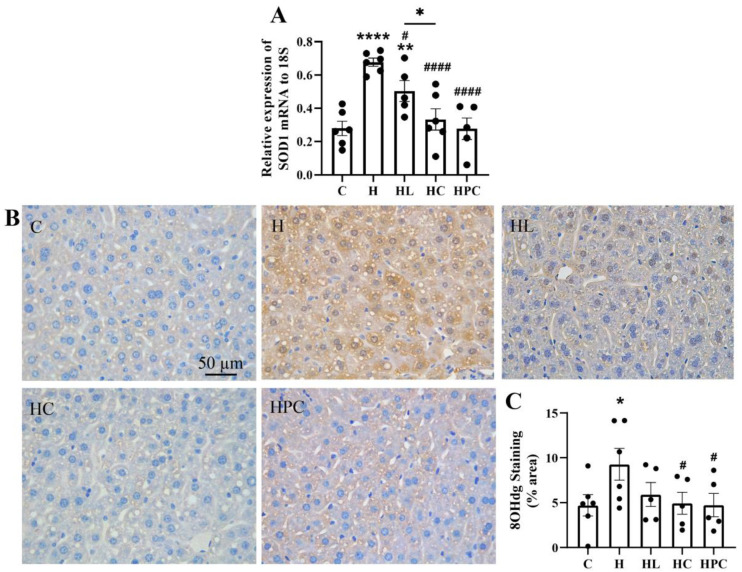
Oxidative stress in the liver of twelve-week-old offspring. (**A**) mRNA expression of antioxidant markers SOD1, (**B**) representative images of 8-OHdg immunohistochemistry at 20× magnification, and (**C**) area (%) of 8-OHdg staining (*n* = 5–6). Results are expressed as mean ± SEM. * *p* < 0.05 ** *p* < 0.01, **** *p* < 0.0001 when compared to C. # *p* < 0.05, #### *p* < 0.0001 when compared to H.

The offspring of mothers with obesity had increased gene expression of the antioxidant marker SOD1 in the liver when compared to the offspring of lean mothers (C vs. H, *p* < 0.0001, Figure 4A). The offspring of mothers with liraglutide or dietary intervention in the perinatal period had reduced expression of SOD1 compared to the offspring of the mothers with obesity (H vs. HL, *p* < 0.05, H vs. HC, H vs. HPC, *p* < 0.0001, Figure 4A). There was a significant decrease in the expression of SOD1 in the offspring of mothers with pre-pregnancy dietary intervention compared to the offspring of mothers with pre-pregnancy liraglutide treatment (HC vs. HL, *p* < 0.05, Figure 4A). 

Oxidative stress levels, as measured by 8-OHdg protein expression, were increased in the liver of offspring of mothers with obesity compared to the offspring of lean mothers (C vs. H, *p* < 0.05, Figure 4B,C). This expression was significantly reduced in the offspring of mothers with perinatal weight intervention via diet change (H vs. HC, H vs. HPC, *p* < 0.05, Figure 4B,C). There was no difference in 8-OHdg protein expression between the offspring of mothers with pre-pregnancy diet change and the offspring of mothers administered pre-pregnancy liraglutide (HC vs. HL, N.S, Figure 4B,C).

### 3.4. Fibrotic Levels in the Liver of Offspring Are Increased Due to Maternal Obesity and Restored by Weight Loss in the Perinatal Period

**Figure 5:**
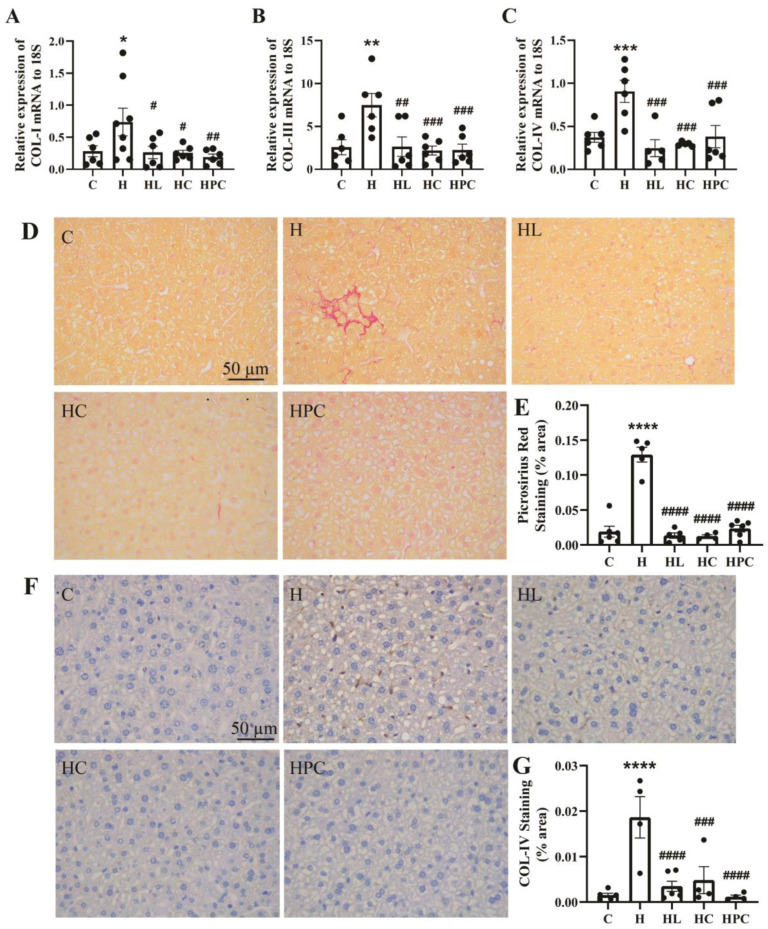
Fibrosis in the liver of twelve-week-old offspring. mRNA expression of fibrotic markers in the liver (**A**) COL-I, (**B**) COL-III, and (**C**) COL-IV (*n* = 6–8). (**D**) Representative images of picrosirius red staining at 20× magnification, (**E**) area (%) of picrosirius red staining (*n* = 5–6), (**F**) representative images of COL-IV immunohistochemistry at 20× magnification, and (**G**) area (%) of COL-IV staining (*n* = 5–6). Results are expressed as mean ± SEM. * *p* < 0.05, ** *p* < 0.01, *** *p* < 0.001, **** *p* < 0.0001 when compared to C. # *p* < 0.05, ## *p* < 0.01, ### *p* < 0.001, #### *p* < 0.0001 when compared to H.

The offspring of mothers with obesity had significantly raised gene expression of COL-I, III, and IV in the liver when compared to the offspring of lean mothers (COL-I: C vs. H *p* < 0.05. COL-III and IV: C vs. H *p* < 0.01, Figure 5A–C). Compared to the offspring of mothers with obesity, the mRNA expression of COL-I, III, and IV was reduced in the offspring of mothers with weight intervention in the perinatal period regardless of the intervention type (COL-I: H vs. HL, HC *p* < 0.05, H vs. HPC *p* < 0.01. COL-III: H vs. HL, HC, HPC *p* < 0.01. COL-IV: H vs. HL, HC *p* < 0.001, H vs. HPC *p* < 0.01, Figure 5A–C). The accumulation of collagens was assessed using picrosirius red staining; the offspring of mothers with obesity without perinatal intervention had increased accumulation of collagens compared to the offspring of lean mothers (C vs. H *p* < 0.0001, Figure 5D,E). The offspring of HFD-fed mothers had increased protein expression of COL-IV when compared to the offspring of lean mothers (C vs. H *p* < 0.0001, Figure 5F,G). Following the same trend as COL-IV mRNA expression, COL-IV protein expression was reduced in the offspring of all mothers with weight intervention in the perinatal period (H vs. HL, HPC *p* < 0.0001, H vs. HC *p* < 0.001, Figure 5F,G). There was no significant difference in the fibrotic phenotype in the liver of offspring of mothers with pre-pregnancy diet intervention compared with the offspring of mothers with pre-pregnancy liraglutide treatment (HC vs. HL, N.S, Figure 5A–G).

## 4. Discussion

The findings from this study contribute to the growing body of evidence demonstrating that maternal obesity has a detrimental effect on the offspring’ risk of chronic disease. Specifically, this study found that maternal obesity programs adverse liver health outcomes for the offspring at late adolescence (week 12), with functional and structural changes seen in the liver consistent with the known histopathologic changes in MAFLD. Importantly, the offspring in this study were weaned to an HFD, which mimics the obesogenic environment frequently experienced by the offspring of mothers with obesity. Male offspring were assessed in this study as it has been shown that they are disproportionately affected by the developmental programming induced by maternal obesity compared to female offspring [16,17,18]. The major finding of this study is that maternal weight intervention in the perinatal period was protective against the adverse liver outcomes for the male offspring of mothers with obesity even when they were raised in an obesogenic environment. For the first time, this study compared the impact of maternal weight loss via dietary intervention versus the administration of GLP-1RA, liraglutide, in ameliorating liver health outcomes in the offspring while comparing the impact of pre-pregnancy diet modification versus diet intervention in early pregnancy. Strikingly, we found that perinatal weight modulation, regardless of the method or timing, improved overall liver health outcomes for the offspring, including reduced liver steatosis, inflammation, oxidative stress, and fibrosis. 

Maternal obesity is a key risk factor for MAFLD in adult offspring. This study confirmed that HFD-fed offspring of obese mothers have phenotypic features of MAFLD including structural, inflammatory, and oxidative stress and fibrotic changes in the liver that were not present in offspring reared from healthy weight mothers. In other rodent models of maternal obesity, this has been attributed to a dysmetabolic and insulin-resistant phenotype [19]. Concordantly, we found that offspring of mothers with obesity had higher levels of insulin resistance, which mechanistically leads to adipose tissue lipolysis and downstream augmentation of lipid regulators [20], contributing to hepatic fat accumulation. Hyperinsulinemia contributed to the upregulation of SREBP-1c in the liver, with overexpression leading to lipogenesis and MAFLD-related steatosis [21]. Previous studies have linked high SREBP-1c expression to in utero exposure to overnutrition [11]. Furthermore, chronic HFD consumption in Japanese macaque mothers was shown to increase hepatic steatosis in the liver of adult offspring, a relationship that was regulated by increased insulin signalling [10]. Protection against hyperinsulinemia and insulin resistance was seen in the offspring of mothers with dietary change in the prenatal period or pharmacological intervention with liraglutide. Equally as effective was early pregnancy dietary intervention. Therefore, our results suggest that a pre-pregnancy or early pregnancy reduction in gestational overnutrition ameliorates offspring insulin resistance in adulthood and reduces adverse foetal programming effects on liver health.

MASH is recognised as an inflammatory disorder which is worsened by systemic inflammation. Serum levels of the inflammatory mediator MCP-1 were unsurprisingly consistent between the offspring groups due to the known upregulation of serum inflammatory markers by chronic HFD feeding in C57Bl6 mice [22]. The upregulation of MCP-1 expression in the liver of offspring of HFD-fed mothers, however, suggests that maternal obesity induces a preferential inflammatory effect to the liver. In a murine model of MCP-1 deficiency, steatosis was ameliorated, suggesting a role in hepatic triglyceride formation [23]. Steatosis concurrent with inflammation can trigger oxidative stress, which was confirmed in the offspring of mothers with obesity as indicated by increased protein and gene expression of 8-OHdg in the liver. Altered cellular programming leading to oxidative stress has been reported as a mechanism of the developmental programming of metabolic dysfunction in the offspring of HFD-fed mothers [24]. Excessive reactive oxygen species (ROS), resultant from oxidative stress, can cause cumulative oxidative damage, and it has been suggested that maternal HFD feeding induces a maternal redox state that affects placental function, altering foetal antioxidant defences [25]. Furthermore, the increase in the antioxidant SOD1 seen in the liver of the offspring of obese mothers in this study indicates increased oxidative stress, as SOD1 scavenges to rebalance ROS. Liver inflammation and oxidative stress act synergistically to induce fibrosis, the ultimate irreversible scarring of the liver. The deposition of extracellular matrix proteins, such as collagens, has been repeatedly reported in the liver of rodents with MASH [26,27], which is consistent with the results from this study, as the upregulation of COL-I, COL-III, and COL-IV was observed. 

AST and ALT are commonly used indicators of liver dysfunction as their elevation is associated with hepatocyte damage [28]. Although this study reported findings of liver dysfunction in the offspring dependent on the maternal diet, there was no difference in AST or ALT levels. Interestingly, this has been previously reported, with other rodent studies finding liver damage in the absence of altered AST and ALT levels at 12–14 weeks of age [29,30], suggesting that these biomarkers may be a late indicator of severe damage not yet present in late adolescence.

A promising perspective emerging from this study is that dietary intervention, even in early gestation, is effective in reducing liver perturbations in the offspring. Our results suggest that metabolic dysfunction is established in utero and therefore interventions in the peri-conception period can be effective at reducing the development of disrupted liver functionality in the individuals exposed to maternal obesity. Accordingly, the downstream effects of metabolic dysfunction and MAFLD were averted in these offspring; the levels of inflammation (MCP-1), oxidative stress (8-OHdg), and fibrosis (COL-I, -III, -IV) in the liver were attenuated by maternal dietary intervention. The results from this study suggest that maternal nutrition, especially in early gestation and pre-pregnancy, regulates the foetal programming of metabolic liver health in the offspring; this supports the hypothesis that adverse effects on offspring liver from maternal obesity can be averted through periconception weight intervention. 

This study aimed to determine the most effective method and timing of maternal weight intervention to facilitate optimal offspring liver health. The literature surrounding the timing of peri-partum weight intervention in the setting of obesity is controversial and scarce. It was reported that dietary intervention from 5 weeks pre-pregnancy was insufficient in preventing diet-induced obesity and insulin resistance in the offspring of HFD-fed dams [31]. Instead, in our study we found that a similar time period reduced clinical features of metabolic dysfunction and limited weight gain and insulin resistance in the same model. Congruous with our results, Zambrano et al. [32] found that dietary intervention from 4 weeks pre-pregnancy was successful in reducing insulin resistance and visceral fat mass in the mouse offspring at 17 weeks of age. Although these studies did not assess the liver health outcomes in the offspring, the latter study found improved insulin resistance, a major component of metabolic dysfunction and indicator of MAFLD risk. We found evidence that suggests that maternal dietary intervention from 5 weeks pre-pregnancy, and even as late as post-conception, had positive impacts on the offspring liver health.

By facilitating meaningful pre-pregnancy maternal weight loss and reduced insulin resistance, liraglutide administration was expected to have the most significant impact on the offspring’s liver health. However, we found that maternal weight intervention through dietary intervention was as effective as liraglutide in facilitating improved offspring liver health outcomes. Overall, the inflammatory and fibrotic indicators of MAFLD were improved by both modes of pre-pregnancy weight loss. The maternal liraglutide group was HFD-fed with concurrent prenatal liraglutide administration. Due to its contraindication during pregnancy [33], liraglutide treatment was ceased at one week prior to pregnancy while an HFD was maintained. This resulted in a higher rate of GWG in this cohort of mothers following conception after the liraglutide was taken away and a mean body weight similar to that of their HFD-fed counterparts in late gestation, as we have previously reported [15]. A birth cohort study found that GWG was associated with an increase in adverse perinatal outcomes [34]. Therefore, GWG could account for the modest differences between the liraglutide and dietary intervention groups. Nonetheless, the offspring of liraglutide-treated mothers had vastly improved liver health outcomes despite higher GWG, indicating that the early gestational period is crucial in the foetal programming of liver health in the offspring.

Maternal health and wellbeing should be considered when interpreting the results of this study to draw conclusions that synergistically benefit both the mother and the child. Although liraglutide treatment was not protective against GWG, it had numerous benefits for the mothers, with improved maternal metabolic health and fertility [15]. Despite the possible ramifications for heightened GWG, the use of liraglutide as a method of pre-pregnancy weight intervention may be the most suitable for many prospective mothers, as weight reduction through dietary intervention alone is difficult to achieve and sustain [13]. Studies that have utilised prenatal dietary strategies to improve peripartum outcomes in women with obesity have been ineffective, most likely due to inadequate weight reduction, with insufficient power to see meaningful benefits for the mother or the children [35]. The practical benefit of pharmacological therapy in attaining significant weight loss benefits in humans may favour this approach, although our preclinical animal model requires translation to human intervention studies. 

## 5. Conclusions

This study has elucidated the remarkable benefits of maternal weight intervention via peri-conception dietary intervention and pre-pregnancy liraglutide administration in the setting of obesity for the liver health of rodent offspring. Our study provides insights into potential methods to alleviate the intergenerational burden of metabolic dysfunction and liver disease in the offspring of mothers with obesity. As the rates of obesity and MAFLD grow globally, it is imperative to determine whether the developmental programming of its fatty, inflammatory, and fibrotic phenotype can be prevented. Pre-pregnancy liraglutide treatment and peri-partum dietary intervention are equally effective in ameliorating adverse liver health outcomes in the offspring. A promising outcome emergent from this study is that all modes of weight intervention, whether they involve dietary modifications before or during pregnancy or pre-pregnancy liraglutide administration, are effective at ameliorating adverse liver effects of maternal obesity in the offspring.

## Figures and Tables

**Table 1:** Mouse primer sequences.

Target	Forward Sequence (5′-3′)	Reverse Sequence (5′-3′)
18S	CGGCTACCACATACCAAGGAA	GCTGGAATTAACCGCGGCT
COL-I	CCCCGGGACTCCTGGACTT	GCTCCGACACGCCCTCTCTC
COL-III	CCTGGAGCCCCTGGACTAATAG	GCCCATTGCACCAGGTTCT
COL-IV	ATGGCTTGCCTGGAGAGATAGG	TGGTTGCCGTTTGAGTCCTGGA
MCP-1	AGGTCCCTGTCATGCTTCTG	GCTGCTGGTGATCCTCTTGT
SOD1	GGTGAACCAGTTGTGTTGTCAG	ATGAGGTCCTGCACTGGTACAG
SREBP-1c	CATGGATTGCACATTTGAAGAC	GCAGGAGAAGAGAAGCTCTCAG

**Table 2:** Body weight and serum measures in twelve-week-old offspring.

Measure	C	H	HL	HC	HPC
Body weight (g)	27.53 ± 0.66	34.45 ± 0.55 ****	34.52 ± 0.83 ****^,††††^	30.62 ± 0.29 **^,###^	32.93 ± 0.50 ****
Liver mass (% total body weight)	4.11 ± 0.19	4.04 ± 0.18	3.84 ± 0.20	4.03 ± 0.17	3.89 ± 0.15
Insulin (pmol/L)	1.03 ± 0.22	1.92 ± 0.31 **	1.05 ± 0.12 ^##^	0.85 ± 0.11 ^###^	0.96 ± 0.12 ^##^
HOMA-IR score	0.072 ± 0.01	0.148 ± 0.02 ***	0.080 ± 0.01 ^##^	0.053 ± 0.01 ^####^	0.065 ± 0.01 ^###^
Glucose (mmol/L)	10.77 ± 0.60	11.75 ± 0.41	12.37 ± 0.40	11.02 ± 0.68	11.27 ± 0.94
NEFA (mmol/L)	0.42 ± 0.03	0.47 ± 0.03	0.48 ± 0.05	0.42 ± 0.04	0.37 ± 0.02
AST (U/L)	96.46 ± 11.95	107.40 ± 17.20	117.80 ± 17.02	121.10 ± 14.26	106.9 ± 18.68
ALT (U/L)	15.62 ± 2.48	16.92 ± 2.61	19.36 ± 2.06	22.10 ± 5.27	20.98 ± 3.18

Results are expressed as mean ± SEM, *n* = 6. ** *p* < 0.01, *** *p* < 0.001, **** *p* < 0.0001 when compared to C. ^##^ *p* < 0.01, ^###^ *p* < 0.001, ^####^ *p* < 0.0001 when compared to H. ^††††^ *p* < 0.0001 when compared to HC.

## Data Availability

Data are contained within the article.
